# Frequency of Fruit and Vegetable Consumption and the Oral Health-Related Quality of Life among Japanese Elderly: A Cross-Sectional Study from the Kyoto-Kameoka Study

**DOI:** 10.3390/nu9121362

**Published:** 2017-12-15

**Authors:** Hinako Nanri, Yosuke Yamada, Aya Itoi, Emi Yamagata, Yuya Watanabe, Tsukasa Yoshida, Motoko Miyake, Heiwa Date, Kazuko Ishikawa-Takata, Mitsuyoshi Yoshida, Takeshi Kikutani, Misaka Kimura

**Affiliations:** 1 Department of Nutrition and Metabolism, National Institutes of Biomedical Innovation, Health and Nutrition, Tokyo 162-8636, Japan; yamaday@nibiohn.go.jp (Y.Y.); t-yoshida@nibiohn.go.jp (T.Y.); 2 Department of Health and Sports Sciences, Kyoto Gakuen University, Kyoto 621-8555, Japan; miyake@kyotogakuen.ac.jp (M.M.); misaka@kyotogakuen.ac.jp (M.K.); 3 Laboratory of Applied Health Sciences, Kyoto Prefectural University of Medicine, Kyoto 602-8566, Japan; yuwatana@mail.doshisha.ac.jp; 4 Department of Health, Sports and Nutrition, Faculty of Health and Welfare, Kobe Women’s University, Hyogo 650-0046, Japan; aitoi@kwjc.kobe-wu.ac.jp; 5 Faculty of Nursing, Doshisha Women’s College of Liberal Arts, Kyoto 610-0395, Japan; eyamagat@dwc.doshisha.ac.jp; 6 Faculty of Health and Sports Science, Doshisha University, Kyoto 610-0394, Japan; 7 Senior Citizens’ Welfare Section, Kameoka City Government, Kyoto 621-8501, Japan; 8 Department of Data Science, Shiga University, Shiga 522-8522, Japan; heiwa.date@biwako.shiga-u.ac.jp; 9 Department of Nutritional Epidemiology and Shokuiku, National Institutes of Biomedical Innovation, Health and Nutrition, Tokyo 162-8636, Japan; kazu@nibiohn.go.jp; 10 Department of Advanced Prosthodontics, Hiroshima University Graduate School of Biomedical & Health Sciences, Hiroshima 734-8553, Japan; mitsu@hiroshima-u.ac.jp; 11 Division of Rehabilitation for Speech and Swallowing Disorders, Nippon Dental University, Tokyo 184-0011, Japan; kikutani@tokyo.ndu.ac.jp

**Keywords:** fruit, vegetable, the oral health-related quality of life, elderly

## Abstract

Objective: Many previous studies have reported that fruit and vegetable consumption is associated with a reduced risk of various disease, but whether or not their consumption is associated with the oral health-related quality of life (OHRQoL) is unclear. The objective of this study was to examine the association between the frequency of fruit and vegetable consumption and the OHRQoL in elderly subjects by sex. Methods: We analyzed cross-sectional data from a population-based Kyoto-Kameoka Study in 2012 of 3112 men and 3439 women (age ≥ 65 years). The frequencies of fruit and vegetable consumption were assessed using a validated food frequency questionnaire. We evaluated the OHRQoL using the General Oral Health Assessment Index (GOHAI), a self-reported measure designed to assess the oral health problems in old adults. Results: After adjusting for age, body mass index, alcohol, smoking, education, socioeconomic status, history of disease, medication use, mobility disability, and total energy intake, a higher frequency of combined fruit and vegetable consumption showed a significant positive association with the GOHAI score in both men and women (*p*-trend < 0.001 in both sexes). These associations remained significant after adjustment for poor mastication and denture use (*p*-trend all < 0.05 in both sexes). We observed a significant positive association even when the frequencies of fruit or vegetable consumption were analyzed separately (all *p*-trend < 0.05 in both sexes). Conclusions: A higher frequency of fruit and/or vegetable consumption independently showed a strong positive association with the OHRQoL in both men and women. Further prospective studies are needed to confirm these findings.

## 1. Introduction

Oral health is one of the major aspects of overall health and oral diseases are the most common form of chronic disease and are important public health problems because of their prevalence and their impact on individuals and society. Many studies have generally suggested that poor oral health, such as tooth loss and periodontal disease, is associated with a higher risk of cancer, cardiovascular disease (CVD), and stroke [1,2,3,4]. Among the elderly in particular, poor oral health is also associated with an increased risk of aspiration pneumonia, cognitive dysfunction, dementia and reduced dietary intake [5,6]. Thus, the prevention of poor oral health in elderly individuals is an important public health issue.

Recent epidemiological studies have also reported on the importance of assessing the general oral health-related quality of life (OHRQoL), which reflects the subjective oral health condition [7,8,9,10,11], as well as objective oral health condition. OHRQoL is defined as an individual’s assessment of one’s own well-being: including functional factors, psychosocial factors and pain/discomfort factors [12,13]. Previous studies have found that a poor OHRQoL was associated with changes in food selection due to impaired chewing ability and negatively affected the dietary intake [14,15], and thus, Consequently, there is a possibility that low intakes of nutrients (e.g., calcium, vitamin C, vitamin D, and polyunsaturated fatty acid (PUFA)) can increase the risk of various disease [16,17]. Therefore, maintaining a good OHRQoL helps protect the overall oral related or other health among middle-aged and elderly individuals.

Fruits and vegetables supply high levels of vitamin E and C, are sources of phytochemicals that function as antioxidants, and are rich in dietary fiber [18]. Numerous cohort studies have reported that the fruit and vegetable consumption has a preventive effect against cancer and CVD through its effects on inflammation [19,20,21,22]. A recent epidemiological study revealed that a higher consumption of vegetables [23,24,25], foods that are rich in antioxidant vitamins [26,27,28], and dietary fiber [29], was associated with a reduced risk of tooth loss and periodontal disease; however, the results related to the association between fruit consumption and tooth loss have been inconsistent [23,24,25]. A higher intake of food containing antioxidant vitamins and dietary fiber was associated with lower levels of inflammation biomarkers [26,30,31], and these nutrients might play an important role in preventive oral health by reducing the degree of oxidative stress in periodontal tissues [26,27,28]. However, no studies have described the relationship between fruit and vegetable consumption and the total score of the OHRQoL.

The objective of the present cross-sectional study was to examine the association between the frequency of fruit and vegetable consumption and the OHRQoL in Japanese elderly individuals.

## 2. Materials and Methods

### 2.1. Study Participants

The present study was conducted based on the baseline survey of the Kyoto-Kameoka Study, which aims to examine the associations between food intake, nutritional status, physical activity, oral function and Long-Term Care (LTC) insurance for community-dwelling older people in Kameoka City, Kyoto Prefecture, Japan. The participants and methods of the Kyoto-Kameoka Study have been described in detail elsewhere [32]. The source population of individuals aged ≥65 years comprised 19,424 people as of 1 July 2011. A total of 16,474 community-dwelling people without LTC certification were invited to participate, and 12,054 responded (response rate: 73.2%). After excluding 69 people who died, an additional survey was conducted for 11,985 subjects in February 2012, and the number of respondents was 8370 (response rate: 69.8%). After merging the data from the baseline survey with the data from the additional survey, 51 subjects who could not be identified with certainty or with inconsistent reports of sex in the 2 surveys were excluded. Ultimately, 8319 subjects were identified as the additional survey population (valid response rate =69.4%).

In the presented study, we excluded 1768 subjects with any of the following conditions: (i) missing data on the General Oral Health Assessment Index (GOHAI) score (*n* = 239) or ≥1 fruit or vegetable item (*n* = 1398); (ii) subjects with extremely low or high dietary energy intake (*n* = 131), i.e., dietary energy intake <800 or ≥4500 kcal/day for men and <500 or ≥3500 kcal/day for women because such intakes are unrealistic and, hence, of questionable validity [33]. Thus, data from 6551 subjects (3112 men and 3439 women) were included in the final analyses.

We described the study concept in the mail survey, and a response to and return of the questionnaire with the subject’s name was regarded as their consent to participate. The entire study protocol was reviewed and approved by the Ethics Committee of Kyoto Prefectural University of Medicine (RBMR-E-363) and National Institute of Health and Nutrition (NIHN187-3). We conducted the survey based on the Ethical Guidelines for Medical and Health Research Involving Human Subjects in Japan (11 November 2007; 22 December 2014; revised 28 February 2017). The details of informed consent was described previous article [32].

### 2.2. Dietary Assessment

A commonly used and previously validated Japanese food frequency questionnaire (FFQ) was used to assess the dietary intake [34,35,36,37]. In this FFQ, we asked participants to report their frequency to assess the average intakes of 46 foods and beverages (green tea and coffee) items over the past year. For the vegetable and fruit items, only frequency options were given, as follows (assigned daily frequencies in parentheses): almost none (0), 1 to 3 times/month (0.1), 1 to 2 times/week (0.2), 3 to 4 times/week (0.5), 5 to 6 times/week (0.8), once/day (1), twice/day (2), and ≥3 times/day (3). The frequency of food items (10 vegetable items and 2 fruits items) consumption was summed for the following foods on the basis of frequency: pumpkin, carrots, broccoli, green leafy vegetables, other green/yellow vegetables, cabbage, *daikon* (Japanese radish), *kiriboshi-daikon*, burdock/bamboo shoot, other vegetable, citrus fruit, and other fruit. We classified the intake frequency of fruits and vegetables into quartiles according to scores. The total energy intake and selected nutrient intakes were calculated using a program developed [34,35], based on the Standard Tables of Food Consumption in Japan (fifth revised edition) [38].

### 2.3. OHRQoL

The GOHAI was developed in the United States by Atchison and Dolan to assess oral health problems in aging populations [33] and is currently the most comprehensive assessment for measuring the OHRQoL [12,32,39,40]. The GOHAI questionnaire have been translated into several languages, and the validated Japanese version was developed by Naito et al. and is now widely used [41,42]. The GOHAI is a questionnaire on the OHRQoL over the past three month. It comprises 12 items grouped into 3 fields: I the functional field (eating, speaking, and swallowing), II the psychosocial field (concerns, relative discomfort, and appearance), and III the pain or discomfort field (drugs, gum sensitivity, and discomfort when chewing certain foods). Available responses to each questionnaire item were as follows: 1, all the time; 2, very often; 3, sometimes; 4, seldom; and 5, never. The cumulative method was used in this study and involved summing the scores obtained for each of the 12 GOHAI questions [33]. Consequently, the GOHAI scale ranged from 12 to 60, with higher scores indicating a good OHRQoL. Generally, a score of 57 to 60 is regarded as high and corresponds to a good oral quality of life (QoL).

### 2.4. Covariates

The mail survey included questions on height, weight, alcohol and smoking status, education, socioeconomic status, history of disease (cardiovascular disease, stroke, cancer, diabetes, hypertension, dyslipidemia, stomach/liver/gallbladder, kidney/prostate), medication use, mobility disability, poor mastication, and denture use. Socioeconomic status was assessed by the following question: “How do you feel about your socioeconomic status?” There were four possible responses: poor, slightly poor, slightly wealthy, and wealthy. The responses were grouped into two categories: (i) poor/slightly poor; or (ii) slightly wealthy/wealthy for this study. Mobility disability was assessed using the Kihon Checklist (KCL) [43,44]. The KCL was developed by the Japanese Ministry of Health, Labor and Welfare to screen for the future risk of requiring long-term care and has been validated for the purpose of screening for frail individuals among the community-dwelling elderly population. The KCL consists of 25 items and has seven domains, including mobility disability (5 items) [43]. The cut-off point for mobility disability was defined as ≥3 points. Each participant’s age was calculated from date of birth on the resident register in the city office. The body mass index (BMI) was calculated as the self-reported body weight (in kg) divided by the square of the self-reported body height (in m).

### 2.5. Statistical Analysis

All analyses were performed separately for men and women because of the sex differences in consumption of fruits and vegetables (Table 1). Differences between men and women in each covariate were evaluated by an unpaired *t*-test for continuous variables or a chi-squared test for categorical variables. The subjects’ characteristics according to frequency of combined fruit and vegetable consumption were assessed by using a linear regression analysis for continuous variables or Mantel-Haenszel test for categorical variables, as appropriate. The adjusted mean values of the GOHAI and their 95% confidence intervals for the fruit and/or vegetable consumption categories were subjected to a general linear regression (GLM) analysis (analysis of covariance). Two different multiple linear regression models were established (Models 1 and 2) to control for potential confounding factors. The first model (Model 1) was adjusted for age (years, continuous), BMI (kg/m^2^, continuous), alcohol consumption status (everyday, sometimes, former, never), smoking status (everyday, sometimes, seldom, never), education (≤9, 10–12, and ≥13 years), socioeconomic status (poor/slightly poor or slightly wealthy/wealthy), history of disease (cardiovascular disease, stroke, cancer, diabetes, hypertension, dyslipidemia, stomach/liver/gallbladder, kidney/prostate; Yes or No), medication use (0–2, 3–4, ≥5), mobility disability (Yes or No), total energy intake (kcal/day, continuous), and fruit consumption (quartiles, for the vegetable analysis) or vegetable consumption (quartiles, for the fruit analysis). The second model (Model 2) was further adjusted for poor mastication (Yes or No) and denture use (Yes or No). All statistical analyses were carried out by using the SAS statistical software package (Ver. 9.4 for Windows; SAS Institute, Cary, NC, USA). *p* values < 0.05 were considered to indicate statistical significance.

## 3. Results

The characteristics of study participants by sex are shown in Table 1. The proportion ≥75 years of age at baseline was 38.0% in men and 39.6% in women. The proportion with BMI ≥ 25.0 kg/m^2^ was 21.4% in men and 19.2% in women. The proportions of participants who were drinkers, smokers, had ≥13 years of education, had a history of disease, and the mean total energy intake were higher in men than in women. In contrast, the proportion of participants with mobility disability, the frequency of fruit and/or vegetable consumption, and the nutrient intake (vitamin E, vitamin C, carotene, dietary fiber) were higher in women than in men.

Table 2 shows the GOHAI scores for the age groups (<75 years or ≥75 years) by sex. The mean GOHAI scores were 53.4 for men <75 years and 51.7 for men ≥75 years and 53.8 for women <75 years and 51.8 for women ≥75 years. The age group of ≥75 years had a lower GOHAI score than that of <75 years in both men and women (*p* values < 0.001, in both sexes).

Table 3 shows the basic characteristics of the study subjects by sex and quartiles of frequency of combined fruit and vegetable consumption. Men who showed a higher frequency of combined fruit and vegetable consumption tended to be older, and include lower proportions of individuals in the following categories: drinker, smoker, poor socioeconomic status, poor mastication, higher proportions of individuals in the following categories: ≥13 years of education, history of disease, and higher intake of total energy and nutrients. BMI did not substantially differ according to combined fruit and vegetable consumption. Women with a higher frequency of combined fruit and vegetable consumption tended to have lower BMI values and to include lower proportions of participants in the following categories: smoker, poor socioeconomic status, medication use ≥5, mobility disability, poor mastication, and denture use, and higher proportions of individuals in the following categories ≥13 years of education, and a higher total energy intake and nutrients.

The adjusted geometric means of the GOHAI and their 95% confidence intervals by quartiles of fruit and/or vegetable consumption are shown in Table 4. The frequency of combined fruit and vegetable consumption was positively associated with the GOHAI after adjustment for age, BMI, alcohol consumption status, smoking status, educational attainment, socioeconomic status, history of disease, medication use, mobility disability, and total energy intake, in both men and women (*p* for trend < 0.001, in both sexes). After adjustment for poor mastication and denture use, these associations were significantly positively associated with the GOHAI score (*p* for trend = 0.006 in men and *p* for trend = 0.02 in women). When separate analyses were conducted for fruit and vegetable consumption, these associations were significantly positively associated with the GOHAI, without adjustment for poor mastication or denture use in men and women (*p* for trend all < 0.001). Even after full adjustment, both fruit consumption and vegetable consumption were significantly positively associated with the GOHAI score in men (fruit consumption, *p* for trend = 0.015; vegetable consumption, *p* for trend = 0.016) and women (fruit consumption, *p* for trend < 0.001; vegetable consumption, *p* for trend = 0.024). We further analyzed the interactions between fruit and/or vegetable consumption and age (<75 years or ≥75 years) on the total GOHAI score but no significant interactions were found (*p* for all interactions > 0.05; data not shown).

## 4. Discussion

To our knowledge, this is the first study to examine the association between fruit and vegetable consumption and the OHRQoL using the GOHAI. Since gender differences in lifestyle and social factors were found, we performed a separate analysis for each sex, and the findings were then adjusted for these confounding factors in our analysis. The frequency of combined fruits and vegetables consumption showed a positive association with the GOHAI. Furthermore, we observed positive association even when the frequencies of fruit and vegetable consumption were analyzed separately. Our findings suggested a strong association between fruit and/or vegetable consumption and the GOHAI as a measure of the OHRQoL in both men and women, even after adjusting for any potentialy confounding factors.

The mean GOHAIs in both men and women of this study were similar to those reported in a previous Japanese study [9,45] but higher than those of studies in other countries [14,46]. Although some studies have reported that older people had a better OHRQoL than young or middle-aged adults [10], many have generally shown that the OHRQoL was inversely associated with age [8,47]. In the present study by age groups, the mean GOHAIs in both sexes were significantly lower in the ≥75-years group than in the <75-years group (Table 2). This may be because clinical disorders, such as tooth loss, occur more frequently in older individuals than in younger ones, so the elderly may have an increasingly higher risk of a poor OHRQoL.

Previous studies have consistently demonstrated an association between the vegetable intake and oral disease [23,24,25]. Wakai et al. found that the consumption of vegetables was positively correlated with the number of remaining teeth among 20,366 Japanese elderly subjects [24]. Brennan et al. found that vegetables (i.e., stir-fried or mixed vegetables, zucchini, eggplant, squash, capsicum, lettuce) were lower risk of tooth loss among older Australian adults [23]. The intakes of dark-green and yellow vegetable were negatively associated with the number of periodontal disease events over a six-year study period [25]. More recent studies have reported that prevention of tooth loss and periodontal disease were associated with reduced risk of a poor OHRQoL [10,11]. Given these findings, our results may suggest that a higher frequency of vegetable intake may have a protective effect on the OHRQoL.

On the other hand, the associations between fruit consumption and oral disease have not been consistent. Some studies have shown no association between fruit consumption and tooth loss [24,25]. Moynihan et al. showed that fruit consumption might be factor in the development of dental caries [48]. In contrast, two studies found that fruit consumption was significantly associated with a reduced risk of oral disease [23,49]. The consumption of specific fruits (i.e., peaches, nectarines, plums, apricots) was associated with a lower risk of tooth loss [23]. Grobler et al. found that the consumption of a high amount of fruits had a beneficial effect on the periodontal status [49]. Our present study supported these findings.

The association observed in the present study may be due in part to the beneficial combination of antioxidant vitamins, such as vitamin C, vitamin E, and polyphenols contained in fruits and vegetables. Among Japanese people, higher intakes of the antioxidants vitamin C, vitamin E, and *β*-carotene were associated with a lower prevalence of periodontal disease [27]. The National Health and Nutrition Examination Surveys (NHANES) III, which included 39,695 subjects, showed a significant relationship between a low vitamin C intake and periodontal disease risk [28]. The human body has several mechanisms to counteract oxidative stress by antioxidants. In one such mechanism, antioxidants act as free radical scavengers by preventing and repairing the damage caused by reactive oxygen species (ROS) and can enhance the immune defenses and reduce the risk of inflammatory disease [50,51]. Lower intake of antioxidant vitamins and polyphenols were associated with higher levels of inflammatory [26], and might play a role in preventive oral health by reducing the oxidative stress in periodontal tissues [25,26,27].

There have been some reports that high-fiber foods such as fruits and vegetables can help protect the oral health [29,30,31]. A previous intervention study reported that high-fiber improved periodontal disease [29]. The odds ratio for an increased CRP concentration (>3.0 mg/L) was 0.49 for the highest quintile of fiber intake compared with the lowest quintile, according to data from the NHANES [30]. Furthermore, higher consumption of chewing-intensive and high-fiber meals led to a reduction in halitosis [52].

In our study subjects, a higher frequency of combined fruit and vegetable consumption was significantly positively associated with the antioxidant nutrient and the dietary fiber intake (Table 3) and was positively correlated with vitamin C (*r* = 0.79 in men and *r* = 0.80 in women), vitamin E (*r* = 0.80 in men and *r* = 0.79 in women), carotenoid (*r* = 0.52 in men and *r* = 0.50 in women), and dietary fiber intake (*r* = 0.80 in both sexes) in men and women (data not shown). These findings suggest that the positive association between higher fruits and/or vegetables and the OHRQoL might be the effect of antioxidant nutrients and dietary fiber.

This study has several methodological limitations. First, our study had a cross-sectional study design, so no temporal relationship between the consumption of fruits and vegetables and the OHRQoL can be inferred, reverse causation might need to be considered for the observed associations. However, similar results were obtained even when participants who showed health-conscious behavior (i.e., selecting healthier foods and supplements; data not shown) were excluded. Moreover, our dietary data was collected only one point in time, lifestyle behaviors such as the dietary intake could not capture longitudinal changes. *Longitudinal studies are required to confirm *these findings.** Second, information was obtained by self-administered questionnaire, so measurement errors were accordingly unavoidable. While the FFQ inquires throughout the diet over the previous year, some degree of seasonal variation in food products might not be able to be completely ruled out. In addition, not only total fruit and vegetable consumption, but also the consumption of a variety of fruits and vegetables could be important for the OHRQoL because consuming various fruits and vegetables provides many different micronutrients and bioactive compounds [53]. In the present study, the average frequency of vegetable or fruit intake was assessed using FFQ; in particular, the frequency of fruit intake was combined from only 2 items (citrus fruit, and other fruit). Thus, the association between OHRQoL and the consumption of various fruits and vegetables remains unclear in the present study and further studies are needed. Third, the results of this study might be affected by potential confounders. For example, a reduced chewing ability or denture use might has been associated with a poor OHRQoL. However, the results of this study did not change the association, even after adjustment for poor mastication and denture use (Table 4). In addition, the socioeconomic status such as the income level and education level may also be associated with OHRQoL. Unfortunately, we did not obtain any information regarding the income level, however, we did obtain data regarding the education level. Because we used a self-reported questionnaire regarding the subjects wealth, the influence of some residual confounding factors on the findings in the present study cannot be ruled out.

## 5. Conclusions

The present cross-sectional study suggest that higher frequencies of consuming fruits and vegetables were positively associated with the OHRQoL in both men and women. Further prospective studies are needed to confirm these findings.

## Figures and Tables

**Table 1:** Characteristics of the study subjects.

	Men	Women	*p* Value ^a^
	(*n* = 3112)	(*n* = 3439)	
Age (years) ^b^	73.4 ± 5.8	73.9 ± 6.2	0.002
<75	1928 (62.0)	2076 (60.4)	0.188
≥75	1184 (38.0)	1363 (39.6)	
Body mass index (kg/m^2^) ^c^	23.1 ± 2.9	22.4 ± 3.3	<0.001
<18.5	85 (2.7)	243 (7.1)	<0.001
18.5–24.9	2350 (75.9)	2508 (73.7)	
≥25.0	663 (21.4)	653 (19.2)	
Current drinker, *n* (%) ^d^	1991 (66.4)	680 (20.4)	<0.001
Current smoker, *n* (%) ^e^	560 (18.6)	118 (3.6)	<0.001
Education (years), *n* (%) ^f^			
≤9	734 (26.2)	885 (28.6)	<0.001
10–12	1198 (42.7)	1665 (53.7)	
≥13	872 (31.1)	549 (17.7)	
Socioeconomic status, *n* (%) ^g^			
Poor	1972 (65.2)	2106 (64.4)	0.50
Wealthy	1053 (34.8)	1165 (35.6)	
History of disease, *n* (%) ^h^	1971 (63.3)	1965 (57.1)	<0.001
Medication use, *n* (%) ^i^			
0–2	1560 (53.7)	1749 (54.8)	0.14
3–4	731 (25.2)	827 (25.9)	
≥5	614 (21.1)	615 (19.3)	
Mobility disability (KCL cut-off ≥3), *n* (%) ^j^	415 (14.3)	817 (26.0)	<0.001
Oral status			
Poor mastication ^k^, *n* (%)	947 (31.1)	1045 (30.9)	0.87
Denture use ^l^, *n* (%)	1735 (57.0)	1996 (59.3)	0.06
Total fruit and vegetable (times/day)	3.5 (2.3, 5.2)	4.6 (3.2, 6.5)	<0.001
Fruit (times/day)	0.6 (0.2, 1.1)	1.0 (0.4, 1.6)	<0.001
Vegetable (times/day)	2.8 (1.8, 4.2)	3.6 (2.4, 5.1)	<0.001
Total energy intake (kcal/day)	1959 ± 449	1576 ± 334	<0.001
Nutrients ^m^			
Vitamin E (mg/day)	9.2 (9.1–9.3)	11.3 (11.2–11.4)	<0.001
Vitamin C (mg/day)	89.0 (87.3–90.6)	123.1 (121.5–124.6)	<0.001
Carotene (µg/day)	2429 (2391–2466)	2683 (2647–2718)	<0.001
Dietary fiber (g/day)	10.1 (9.9–10.2)	11.5 (11.4–11.7)	<0.001

KCL, Kihon Checklist. Values are mean ± standard deviation or median (25%, 75%) for continuous variables and number (%) for categorical variables. ^a^ *p* values are based on unpaired *t*-test or Wilcoxon rank-sum test for continuous variables and chi-squared tests for categorical variables. ^b^ Based on 3112 men and 3439 women. ^c^ Based on 3098 men and 3404 women. ^d^ Based on 3000 men and 3330 women. ^e^ Based on 3007 men and 3296 women. ^f^ Based on 2804 men and 3099 women. ^g^ Based on 3025 men and 3271 women. ^h^ Based on 3112 men and 3439 women. ^i^ Based on 2905 men and 3191 women. ^j^ Based on 2895 men and 3143 women. ^k^ Based on 3043 men and 3378 women. ^l^ Based on 3043 men and 3364 women. ^m^ Adjusted for total energy intake.

**Table 2:** Mean General Oral Health Assessment Index (GOHAI) score by sex and age group.

	Total	Age Group (In Years)		*p* Value ^a^
		<75	≥75	
Men, *n*	3112	1928	1184	
	52.7 ± 8.1	53.4 ± 7.7	51.7 ± 8.5	<0.001
Women, *n*	3439	2076	1363	
	53.0 ± 7.9	53.8 ± 7.4	51.8 ± 8.5	<0.001

GOHAI, Geriatric Oral Health Assessment Index. Mean ± standard deviation. ^a^ *p* values are based on unpaired *t*-test.

**Table 3:** Characteristics of the study subjects according to categories of frequency of combined fruit and vegetable consumption.

	Q1 (Lowest)	Q2	Q3	Q4 (Highest)	*p* Value ^a^
Men, *n* (%)	762 (24.5)	779 (25.0)	798 (25.6)	773 (24.9)	
Total fruit and vegetable (times/day)	1.59 (1.49–1.70)	2.89 (2.79–2.99)	4.30 (4.20–4.40)	7.60 (7.50–7.71)	<0.001
Fruit (times/day)	0.27 (0.23–0.32)	0.54 (0.50–0.59)	0.86 (0.81–0.90)	1.48 (1.44–1.52)	<0.001
Vegetable (times/day)	1.32 (1.22–1.42)	2.35 (2.25–2.45)	3.44 (3.35–3.54)	6.13 (6.03–6.22)	<0.001
Age (years) ^b^	72.9 ± 5.5	72.9 ± 5.8	73.3 ± 5.7	74.5 ± 5.9	<0.001
Body mass index (kg/m^2^) ^c^	23.1 ± 2.9	23.1 ± 2.8	23.1 ± 3.1	23.0 ± 2.7	0.60
Current drinker, *n* (%) ^d^	505 (68.3)	509 (68.1)	525 (68.4)	452 (60.6)	0.002
Current smoker, *n* (%) ^e^	183 (24.7)	147 (19.6)	127 (16.4)	103 (13.8)	<0.001
Education ≥ 13 years, *n* (%) ^f^	165 (24.6)	224 (31.2)	236 (32.9)	247 (35.5)	<0.001
Poor socioeconomic status, *n* (%) ^g^	543 (71.9)	502 (65.7)	486 (63.4)	450 (59.9)	<0.001
History of disease, *n* (%) ^h^	459 (60.2)	490 (62.9)	511 (64.0)	66.1 (66.1)	0.016
Medication use ≥ 5, *n* (%) ^i^	147 (20.5)	138 (19.2)	156 (20.7)	173 (24.2)	0.15
Mobility disability, *n* (%) ^j^	111 (16.0)	113 (15.2)	96 (12.8)	95 (13.4)	0.08
Poor mastication, *n* (%) ^k^	287 (38.7)	220 (28.7)	228 (29.1)	212 (28.3)	<0.001
Denture use, *n* (%) ^l^	451 (61.0)	412 (53.5)	446 (57.0)	426 (56.7)	0.25
Total energy intake (kcal/day)	1867 ± 454	1915 ± 409	1965 ± 428	2087 ± 473	<0.001
Vitamin E (mg/day) ^m^	7.1 (6.9–7.3)	8.6 (8.36–8.73)	10.0 (9.8–10.1)	13.2 (13.0–13.4)	<0.001
Vitamin C (mg/day) ^m^	62.4 (60.3–64.6)	80.8 (78.7–82.9)	97.6 (95.6–99.7)	137.9 (135.7–140.0)	<0.001
Carotene (µg/day) ^m^	2030 (1963–2096)	2326 (2261–2392)	2569 (2504–2633)	3183 (3117–3250)	<0.001
Dietary fiber (g/day) ^m^	8.2 (8.1–8.4)	9.5 (9.3–9.7)	10.9 (10.8–11.1)	14.2 (14.0–14.3)	<0.001
Women, *n* (%)	881 (25.6)	798 (23.2)	908 (26.4)	852 (24.8)	
Total fruit and vegetable (times/day)	2.30 (2.19–2.42)	3.90 (3.78–4.02)	5.48 (5.37–5.59)	9.28 (9.17–9.40)	<0.001
Fruit (times/day)	0.45 (0.40–0.50)	0.86 (0.81–0.91)	1.23 (1.18–1.28)	1.97 (1.92–2.02)	<0.001
Vegetable (times/day)	1.85 (1.74–1.96)	3.04 (2.93–3.15)	4.25 (4.14–4.35)	7.32 (7.21–7.41)	<0.001
Age (years) ^b^	74.1 ± 6.6	73.5 ± 6.0	73.5 ± 6.0	74.5 ± 5.9	0.32
Body mass index (kg/m^2^) ^c^	22.7 ± 3.5	22.5 ± 3.2	22.4 ± 3.3	22.2 ± 3.1	0.001
Current drinker, *n* (%) ^d^	195 (22.8)	150 (19.6)	169 (19.2)	166 (20.1)	0.25
Current smoker, *n* (%) ^e^	55 (6.5)	27 (3.6)	18 (2.1)	18 (2.2)	<0.001
Education ≥13 years, *n* (%) ^f^	97 (12.8)	116 (16.0)	155 (18.5)	181 (23.4)	<0.001
Poor socioeconomic status, *n* (%) ^g^	583 (69.4)	503 (66.6)	538 (62.2)	482 (59.4)	<0.001
History of disease, *n* (%) ^h^	514 (58.3)	451 (56.5)	508 (56.0)	492 (57.8)	0.73
Medication use ≥5, *n* (%) ^i^	181 (22.6)	144 (19.5)	156 (18.1)	134 (16.9)	0.001
Mobility disability, *n* (%) ^j^	268 (34.0)	178 (24.4)	193 (22.9)	178 (22.8)	<0.001
Poor mastication, *n* (%) ^k^	317 (36.9)	252 (32.1)	261 (29.2)	215 (25.6)	<0.001
Denture use, *n* (%) ^l^	529 (61.7)	483 (61.2)	500 (56.4)	484 (58.2)	0.04
Total energy intake (kcal/day)	1473 ± 343	1527 ± 296	1580 ± 298	1725 ± 340	<0.001
Vitamin E (mg/day) ^m^	8.0 (7.8–8.2)	9.6 (9.4–8.2)	11.2 (11.0–11.4)	14.5 (14.3–14.7)	<0.001
Vitamin C (mg/day) ^m^	79.5 (77.2–81.8)	101.7 (99.3–104.0)	123.0 (120.7–125.2)	167.1 (164.7–169.4)	<0.001
Carotene (µg/day) ^m^	2158 (2097–2219)	2,391 (2327–2454)	2641 (2581–2700)	3176 (3113–3239)	<0.001
Dietary fiber (g/day) ^m^	8.6 (8.5–8.8)	9.9 (9.7–10.0)	11.2 (11.0–11.3)	14.1 (14.0–14.3)	<0.001

Q, quartile of frequency of combined fruit and vegetable consumption; Q1, <25th percentile; Q2, ≥25th percentile and <50th percentile; Q3, ≥50th percentile and <75th percentile; Q4, ≥75th percentile. Values are median (25%, 75%) or mean ± standard deviation for continuous variables and number (%) for categorical variables. ^a^ *p* values for linear trends across quintiles are based on linear regression analysis for continuous variables and the Mantel-Haenszel chi-squared tests for categorical variables. ^b^ Based on 3112 men and 3439 women. ^c^ Based on 3098 men and 3404 women. ^d^ Based on 3000 men and 3330 women. ^e^ Based on 3007 men and 3296 women. ^f^ Based on 2804 men and 3099 women. ^g^ Based on 3025 men and 3271 women. ^h^ Based on 3112 men and 3439 women. ^i^ Based on 2905 men and 3191 women. ^j^ Based on 2895 men and 3143 women. ^k^ Based on 3043 men and 3378 women. ^l^ Based on 3043 men and 3364 women. ^m^ Adjusted for the total energy intake.

**Table 4:** Adjusted geometric means (95% confidence interval) of GOHAI score by quartiles of frequency of fruit and vegetable consumption in study subjects.

		Q1 (Lowest)	Q2	Q3	Q4 (Highest)	*p* for Trend ^a^
Men						
Combined fruit and vegetable	*n* (%)	762 (24.5)	779 (25.0)	798 (25.6)	773 (24.9)	
Model 1 ^b^		51.8 (51.2–52.4)	52.8 (52.2–53.4)	53.6 (53.0–54.2)	53.4 (52.8–54.1)	<0.001
Model 2 ^c^		52.3 (51.7–52.9)	52.7 (52.2–53.3)	53.4 (52.9–54.0)	53.2 (52.7–53.8)	0.006
Fruit	*n* (%)	871 (28.0)	814 (26.2)	682 (21.9)	745 (23.9)	
Model 1 ^d^		52.1 (51.5–52.7)	52.6 (52.0–53.2)	53.3 (52.7–54.0)	53.8 (53.2–54.5)	<0.001
Model 2 ^e^		52.4 (51.9–53.0)	52.8 (52.3–53.4)	53.2 (52.6–53.8)	53.4 (52.8–53.9)	0.015
Vegetable	*n* (%)	794 (25.5)	730 (23.5)	821 (26.4)	767 (24.7)	
Model 1 ^f^		52.0 (51.4–52.7)	52.7 (52.0–53.3)	53.6 (53.0–54.2)	53.3 (52.7–54.0)	<0.001
Model 2 ^g^		52.4 (51.8–52.9)	52.8 (52.2–53.3)	53.4 (52.9–53.9)	53.2 (52.6–53.8)	0.016
Women						
Combined fruit and vegetable	*n* (%)	881 (25.6)	798 (23.2)	908 (26.4)	852 (24.8)	
Model 1 ^b^		52.2 (51.6–52.8)	53.1 (52.5–53.8)	53.7 (53.1–54.2)	53.8 (53.2–54.5)	<0.001
Model 2 ^c^		52.6 (52.0–53.1)	53.3 (52.8–53.9)	53.6 (53.1–54.1)	53.5 (52.9–54.0)	0.020
Fruit	*n* (%)	1,018 (29.6)	899 (26.1)	764 (22.2)	758 (22.0)	
Model 1 ^d^		51.9 (51.3–52.5)	53.5 (52.9–54.1)	54.0 (53.4–54.7)	53.7 (53.1–54.4)	<0.001
Model 2 ^e^		52.3 (51.8–52.8)	53.5 (53.0–54.1)	53.9 (53.3–54.4)	53.5 (52.9–54.1)	<0.001
Vegetable	*n* (%)	817 (23.8)	906 (26.3)	889 (25.9)	827 (24.1)	
Model 1 ^f^		52.3 (51.6–52.9)	53.2 (52.6–53.8)	53.6 (53.0–54.2)	53.8 (53.2–54.4)	<0.001
Model 2 ^g^		52.7 (52.2–53.3)	53.1 (52.6–53.6)	53.5 (53.0–54.0)	53.6 (53.0–54.1)	0.024

GOHAI, Geriatric Oral Health Assessment Index. Q, quartile of frequency of combined fruit and vegetable consumption; Q1, <25th percentile; Q2, ≥25th percentile and <50th percentile; Q3, ≥50th percentile and <75th percentile; Q4, ≥75th percentile. ^a^ Trend tests were performed by including the ordinal variable in a linear regression analysis. ^b^ Adjusted for age (years), body mass index (kg/m^2^), alcohol status (everyday, sometimes, seldom, or never), smoking status (everyday, sometimes, seldom, or never), education (≤9, 10–12, and ≥13 years), socioeconomic status, history of disease (hypertension, diabetes, dyslipidemia, cancer, cardiovascular disease, stroke, liver disease, chronic renal failure; Yes or No), medication use (0–2, 3–4, ≥5), mobility disability (Yes or No) and total energy intake (kcal/day). ^c^ Model 1 + poor mastication (Yes or No) and denture use (Yes or No). ^d^ The same as model 1^b^ with adjustment for frequency of vegetable intake (quartiles of vegetable intake). ^e^ Model 1^d^ + poor mastication (Yes or No) and denture use (Yes or No). ^f^ The same as model 1^b^ with adjustment for frequency of fruit intake (quartiles of fruit intake). ^g^ Model 1^f^ + poor mastication (Yes or No) and denture use (Yes or No).

## Abbreviations

OHRQoL	oral health-related quality of life;
GOHAI	general oral health assessment index;
FFQ	food frequency questionnaire;
CVD	cardiovascular disease;
BMI	body mass index
